# Endocan attenuates LPS-induced alveolar type II cells injury through PI3K/Akt/mTOR pathway

**DOI:** 10.48101/ujms.v131.13337

**Published:** 2026-03-04

**Authors:** Zekai Wang, Yulu Zhao, Ming Luo, Jinling Yang, Zhixiang Geng, Shining Ou, Zhenxuan Huang, Ruiqi Sun, Xiang Luo, Keke Jin, Fangyan Wang, Xiaolong Zhang

**Affiliations:** a Department of Pathophysiology, School of Basic Medical Sciences, The Research Institute of Microbiota and Host Inflammation-Related Diseases, Wenzhou Medical University, Wenzhou, China; b The Second Affiliated Hospital and Yuying Children’s Hospital of Wenzhou Medical University, Wenzhou, China; c Department of Anesthesiology, Critical Care and Pain Medicine, The Second Affiliated Hospital and Yuying Children’s Hospital of Wenzhou Medical University, Wenzhou, China; d Department of Chemical and Environmental Engineering, The University of Nottingham Ningbo China, Ningbo, PR China

**Keywords:** Endocan, mTOR, ALI, lipopolysaccharide, alveolar type II cell

## Abstract

**Background:**

Alveolar type II (AT2) cell injury plays an important role in the pathogenesis of acute lung injury (ALI), but the corresponding treatment options are limited in clinical practice. Endocan has been proved to exert a protective effect in ALI, however, the underlying mechanism remains unclear. The phosphoinositide 3-kinase (PI3K)/protein kinase B (AKT)/mechanistic target of rapamycin (mTOR) signaling pathway was found to exhibit beneficial effects in lipopolysaccharide-induced ALI. This study aimed to investigate protective effects of endocan on AT2 cells and the signal pathway in LPS-induced ALI.

**Methods:**

Pulmonary function testing and hematoxylin–eosin staining were employed to evaluate the effects of endocan on the LPS-induced ALI in mice. Transmission electron microscopy (TEM) analysis, immunofluorescence and Western blot were used to assess the AT2 protection of endocan. Mouse lung epithelial cell line 12 (MLE-12) cells were facilitated to observe the activation of PI3K/AKT/mTOR pathway.

**Results:**

Endocan administration effectively ameliorated respiratory parameters in LPS-challenged ALI mice. TEM revealed that endocan treatment preserved AT2 cell integrity and maintained lamellar body ultrastructure compared to the mice injected LPS only. Western blot analysis showed a higher surfactant protein C expression in endocan-treated mice than that of model group. Moreover, the phosph-PI3K, -AKT, -mTOR levels detected by Western blot were significantly observed upregulated after endocan treatment. However, rapamycin, an mTOR inhibitor, abolished the protective effects of endocan against ALI, indicating this pathway may be critical for its action on AT2 cells. In LPS-treated MLE-12 cells, the Western blot analysis further confirmed that rapamycin suppressed endocan-induced activation of the PI3K/AKT/mTOR pathway, thereby attenuating the protective effects of endocan on MLE-12 cells.

**Conclusion:**

Endocan protects AT2 cells against ALI through activating PI3K/AKT/mTOR pathway, suggesting its therapeutic potential for AT2 in patients with ALI.

## Introduction

Acute lung injury (ALI) is characterized by excessive pulmonary inflammation of leukocyte diapedesis, resulting in alveolar epithelial damage and pulmonary edema, which may progress to acute respiratory distress syndrome (ARDS) in severe cases (1–3). Alveolar type II epithelial (AT2) cells produce surfactant and repair damaged cells, playing a pivotal role in maintaining barrier function and preventing fluid leakage in ALI (4, 5). Given that AT2 cell injury substantially contributes to the development of ALI (6), strategies aimed at preserving AT2 cell integrity may represent a potential therapeutic approach.

Endocan, also known as endothelial cell-specific molecule-1, is a novel soluble circulating proteoglycan secreted by endothelial cells (7, 8). Present in the pulmonary microcirculation, endocan is implicated in endothelial dysfunction, inflammation, and altered vascular permeability, making it a potential biomarker and therapeutic target for ALI and ARDS (9–12). Our previous study demonstrated that endocan could significantly suppress inflammatory responses in ALI (13). Additionally, clinical analysis also found that plasma endocan concentrations effectively reflect the severity and prognosis of ARDS (14, 15). However, its effects on AT2 cells in the context of LPS-induced ALI remain underexplored.

The PI3K/AKT/mTOR pathway is a highly conserved network critical for cellular functions, including survival and differentiation (16, 17). In LPS-induced ALI of mice, the PI3K/AKT/mTOR pathway mediates the anti-inflammatory effects of liensinine by modulating autophagy (18). Moreover, Feng et al. demonstrated that Acacia catechu (L.f.) Willd and Scutellaria baicalensis Georgi exerted potent anti-inflammatory effects in LPS-induced ALI in rats by attenuating the NF-κB, Mitogen-activated protein kinase (MAPK), and PI3K-Akt signaling pathways (19). These findings suggest that regulating the PI3K/AKT/mTOR signaling pathway may represent a novel therapeutic strategy for ALI and ARDS.

Our study utilized LPS intratracheal administration to simulate the pathological conditions of ALI and originally focus on the role of AT2 cells in mediating the therapeutic effects of endothelin through activating PI3K/AKT/mTOR pathway. These findings aimed to further reveal a novel mechanism of endocan in ALI and highlight its potential as a therapeutic target for inflammatory lung diseases.

## Materials and methods

### Experimental animals

Male C57BL/6 mice, aged 6–8 weeks, were purchased from Beijing Weitonglihua Experimental Animal Technology Co. Ltd. (Beijing, China). The mice were housed in a Specific pathogen-free (SPF)-grade facility at the Animal Experimental Center of Wenzhou Medical University and provided with a standard diet, free drinking water, and a 12:12 h light-dark cycle.

### Ethical approval

The animal study protocol was approved by the Institutional Animal Care and Use Committee of the Experimental Animal Centre of Wenzhou Medical University (xmsq2024-0691). All methods were carried out in accordance with relevant guidelines and regulations. This study was carried out in compliance with the ARRIVE (Animal Research Reporting In Vivo Experiment) guidelines.

### In vivo LPS-induced lung injury model

Mice were randomly divided into seven groups (*n* = 4): Control, LPS, LPS + Endocan (0.1 mg/kg), LPS + Endocan (0.3 mg/kg), LPS + Endocan (0.5 mg/kg), LPS + Rapamycin (2 mg/kg), and LPS + Endocan (0.3 mg/kg) + Rapamycin (2 mg/kg). Outcome assessors for pulmonary function, histology, and Western blot densitometry were blinded to group allocation. Group size (*n* = 4) was determined based on feasibility and prior pilot experiments, and key experiments were replicated independently at least twice to ensure reproducibility.

Anesthesia was induced using 2% isoflurane, confirmed by muscle relaxation and a lack of response to external stimuli. LPS was injected intratracheally at a dose of 5 mg/kg to establish the mouse model of ALI using a pipette tip for intratracheal instillation. Endocan was administered intraperitoneally 30 min before LPS to model prophylactic intervention, based on previous studies indicating early endothelial targeting maximizes protective effects. The 6-h timepoint was chosen as it represents the critical phase of ALI progression characterized by maximal inflammatory response and alveolar injury (13).

Rapamycin was administered intraperitoneally for three consecutive days prior to LPS challenge in the relevant groups to inhibit mTOR signaling. Post-procedural recovery was facilitated under thermoneutral conditions using temperature-controlled heating pads.

At the 6-h critical phase of ALI progression, euthanasia was performed through intraperitoneal injection of sodium pentobarbital (200 mg/kg), and lung tissues were harvested for subsequent analysis.

### Non-invasive measurement of pulmonary function

Whole Body Plethysmography (WBP) (Tow Intelligent Technology, Shanghai, China) was used to assess pulmonary function. After instrument calibration, mice were placed in the plethysmography box and respiration was monitored continuously. The respiratory parameters measured included frequency (F), peak expiratory flow (PEF), peak inspiratory flow (PIF), Time of Expiration (TE), minute volume (Mv), Time of Inspiration (TI), tidal volume (Vt).

### Hematoxylin–eosin staining

Hematoxylin–eosin (H&E) staining was conducted to examine the lung morphology in mice. The left lung tissues were fixed in 4% paraformaldehyde overnight, followed by dehydration, paraffin embedding, and serial sectioning into 5 μm slices. Subsequently, the sections were stained with H&E, visualized and imaged using an optical microscope (Nikon, Tokyo, Japan).

### Myeloperoxidase (MPO) activity assays

MPO activity assays were conducted on homogenized lung tissues using an MPO Detection Kit (Nanjing Jiancheng Bioengineering Institute, Nanjing, China). The activity was quantified by measuring spectrophotometric absorbance changes at 460 nm and presented as units per gram of total protein (U/g). Total protein concentrations were determined via the Bicinchoninic acid (BCA) protein assay kit (Yamei, ZJ101).

### Western blotting

Total protein samples were extracted from lung tissues using RIPA buffer (Yamei, PC101). Protein concentrations were also assessed using a BCA protein assay kit (Yamei, ZJ101). Proteins were separated by 7.5 and 10% sodium dodecyl sulfate–polyacrylamide gel electrophoresis and transferred to polyvinylidene fluoride membranes. The membranes were blocked in 8% skimmed milk for 2 h and then incubated overnight at 4°C with primary antibodies: surfactant protein C (SP-C) (Invitrogen, PA5-71680), mTOR (Cell Signaling Technology (CST), 2983S), p-mTOR (CST, 5536S), PI3K (CST, 4292S), p-PI3K (CST, 4228S), AKT (CST, 4691S), p-AKT ((CST), 4060S), GAPDH (HUABIO, ET1601-4). After three times washes with Tris-buffered saline, the membranes were incubated with appropriate secondary antibodies at room temperature for 1 h. Imprinting was observed using chemiluminescence (Yamei, SQ201) and an Odyssey imaging system (Li-Cor Biosciences, NE, USA).

### Transmission electron microscopy analysis

Lung tissues were excised and washed with precooled PBS (pH 7.4). The right lung apex was incubated overnight in 2.5% glutaraldehyde for fixation. Selected lung areas were postfixed in 1% osmium tetroxide for 1 h, and dehydrated through a graded acetone series and embedded in epoxy resin. The polymerization was performed at 60°C for 48 h. Ultrathin sections (100 nm) were cut, and stained with uranyl acetate and lead citrate, and viewed under a H-7500 transmission electron microscopy (TEM) (Hitachi, Tokyo, Japan). Five fields were randomly selected for each sample.

### Immunofluorescent staining

Frozen tissue sections were washed in PBS for 5 min and incubated overnight at 4°C with a primary antibody against SP-C (Invitrogen, PA5-71680, 1:200). Following PBS washes, the sections were incubated with a secondary antibody (Proteintech, SA00013-2, 1:500) at room temperature for 1 h. After rinsing three times in PBS, the sections were mounted with 4’,6-diamidino-2-phenylindole (Beyotime Biotechnology, China) and observed under an orthographic fluorescence microscope.

### Cell culture and treatment

mouse lung epithelial cell line 12 (MLE-12) (FuHeng Cell Bank, Shanghai, China) was cultured in DMEM/F12 medium (Procell, Cat# CM-0680) supplemented with 10% fetal bovine serum and 1% penicillin/streptomycin at 37°C with 5% CO2. When cells reached approximately 85% confluence, MLE-12 cells were seeded into 12-well and cultured until adherence. Cells were randomly assigned to six groups: Control, Endocan (30 ng/mL), LPS (10 μg/mL), LPS + Rapamycin (250 nM), LPS + Endocan (30 ng/mL), LPS + Endocan (30 ng/mL) + Rapamycin (250 nM) (20). After 24 h of incubation, cells were collected for protein extraction and Western blot analysis.

### Statistical analysis

GraphPad Prism (version 9.0) was used for statistical analysis. Experimental data were shown as the mean ± standard deviation. The normality of sample distributions was assessed using the Kolmogorov–Smirnov test, and all variables included in parametric analyses met the normality assumption (*P* > 0.05). Two-tailed unpaired Student’s *t*-test and one-way analysis of variance with Tukey’s correction were used for all comparisons of mice-related experiments. *P*-value < 0.05 was considered significant.

## Results

### Endocan attenuated LPS-induced pulmonary function impairment

**Figure 1:**
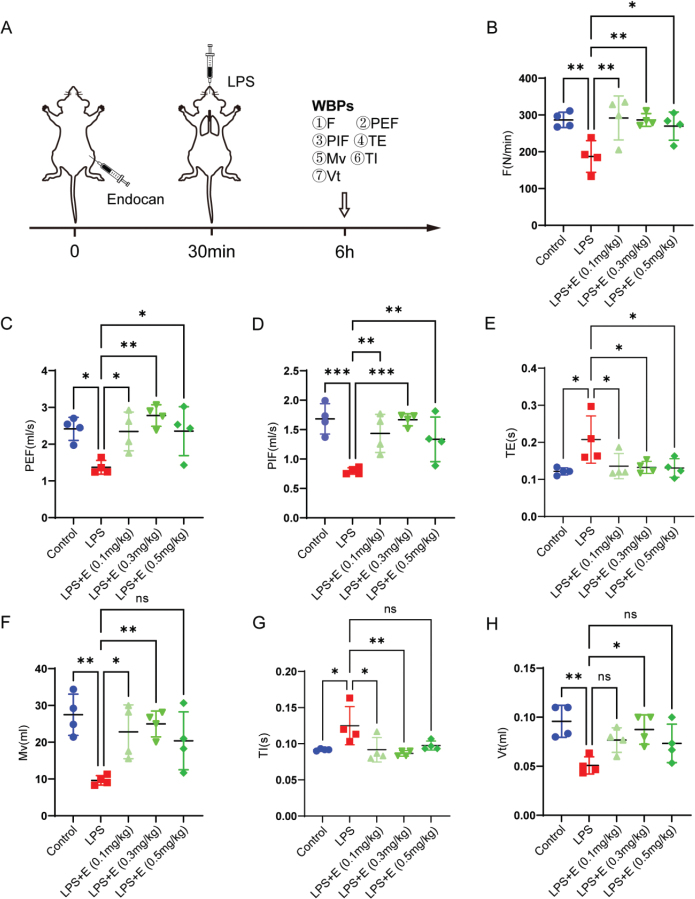
Endocan significantly mitigated LPS-induced respiratory function impairment in mice. (A) Schematic of the LPS-induced lung injury mice model. (B–H) Male C57BL/6 mice were randomized into 5 groups (*n* = 4): Control, LPS, LPS + Endocan (0.1 mg/kg), LPS + Endocan (0.3 mg/kg), and LPS + Endocan (0.5 mg/kg). Pulmonary function parameters, including: B: frequency (F), C: peak expiratory flow (PEF), D: peak inspiratory flow (PIF), E: Time of Expiration (TE), F: minute volume (Mv), G: Time of Inspiration (TI), H: tidal volume (Vt) were assessed 6 h after LPS instillation as described in the Methods. *n* = 4, data are expressed as mean ± SD. One-way analysis of variance (ANOVA) was used to statistical analyze; *P < 0.05, **P < 0.01, ***P < 0.001.

To investigate the protective effects of endocan on pulmonary function, we established an LPS-induced ALI model treated with varying concentrations of endocan prior to LPS administration (Figure 1A). LPS exposure resulted in significant impairment of respiratory parameters, including frequency (F), minute ventilation (Mv), tidal volume (Vt), peak expiratory flow (PEF), and peak inspiratory flow (PIF), as well as prolonged inspiratory time (TI) and expiratory time (TE). Treatment with endocan led to significant improvements in these parameters, indicating a consistent alleviation of LPS-induced pulmonary dysfunction. Notably, given the fact that 0.3 mg/kg dose demonstrated optimal and consistent alveolar protection, we chose this selection as the experimental concentration for subsequent investigations. Collectively, these findings indicate that endocan alleviated LPS-induced respiratory dysfunction, maximal at 0.3 mg/kg.

### Endocan preserved AT2 cells integrity in LPS-induced lung tissues and activated PI3K/AKT/mTOR pathway

**Figure 2:**
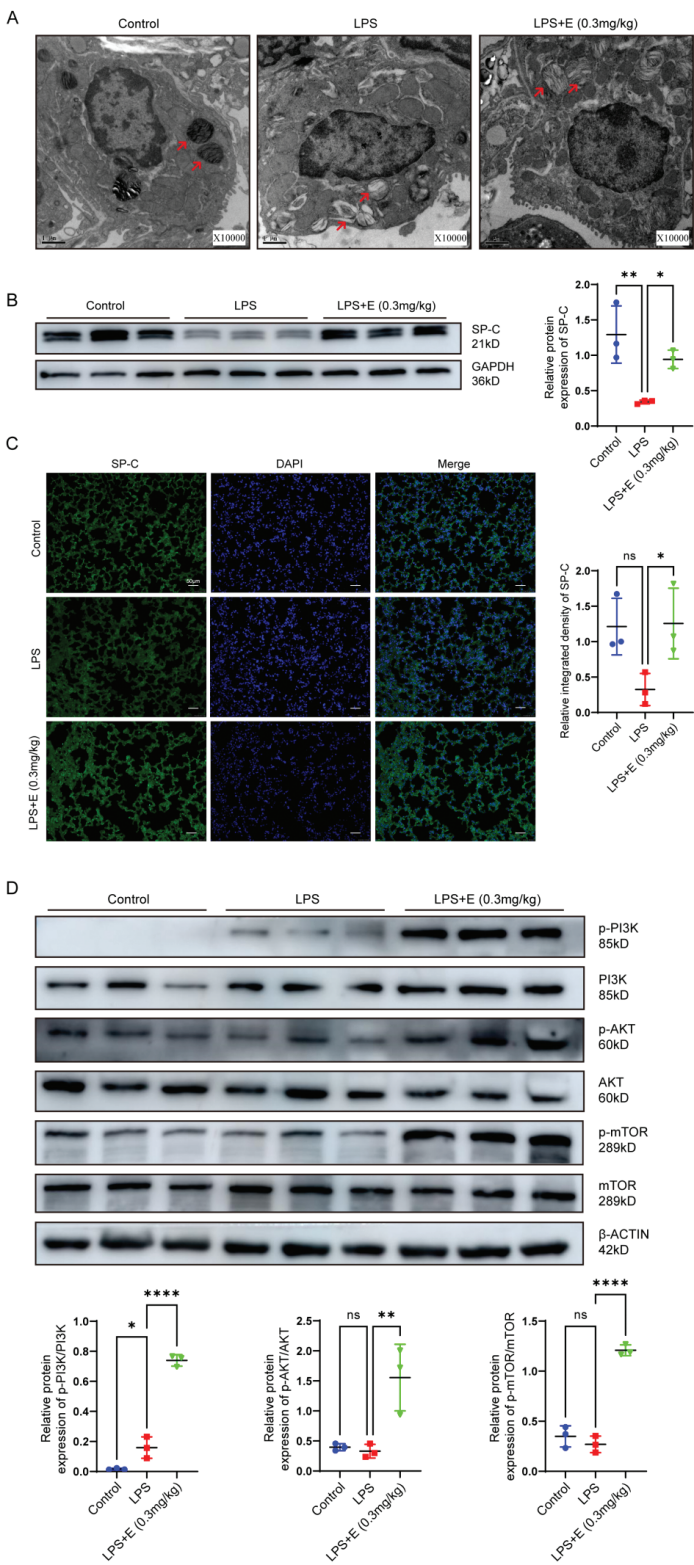
Endocan mitigated LPS-induced morphological and molecular changes in AT2 cells. (A) Transmission electron microscopy (TEM) images illustrate the cellular morphology and the structure of LBs in the control, LPS, and endocan-treated groups in AT2 cells. (B) Western blot analysis showing the expression levels of surfactant protein C (SP-C). (C) Immunofluorescent staining of lung tissues in control, LPS, and endocan-treated groups. (D) Western blot analysis showing the expression levels of PI3K and p-PI3K, Akt and p-Akt, mTOR and p-mTOR in AT2 cells. Scale bar = 50 μm. *n* = 3, data are expressed as mean ± SD. One-way analysis of variance (ANOVA) was used for statistical analyze; *P < 0.05, **P < 0.01, ****P < 0.0001.

AT2 cells play a critical role in the pathophysiology of ALI (21). To investigate the involvement of AT2 cells in ALI-associated pathological changes, we utilized TEM to examine their morphology. In the LPS group, lamellar bodies (LBs), the hallmark organelles of AT2 cells, were significantly enlarged, exhibited vacuolization, and displayed extensive structural damage. Conversely, these pathogenic changes were significantly reversed with endocan treatment (Figure 2A). Additionally, SP-C, a well-established AT2 marker that reflects AT2 activity to some extent (22), was upregulated after endocan administration (Figure 2B). This finding was further supported by immunofluorescence analysis (Figure 2C), strongly indicating that endocan confers protective effects on AT2 cells and has therapeutic potential in mitigating ALI. Existing research suggests that endocan significantly influences cell proliferation (23). Concurrently, mTOR, a serine/threonine protein kinase, is known to regulate cell growth (24). Therefore, we investigated the mTOR signaling pathway. As expected, our results revealed a significant increase in the protein expression levels of p-PI3K, p-AKT, and p-mTOR in lung tissues of endocan-treated mice compared to the LPS group (Figure 2D). These findings suggest that endocan effectively mitigates LPS-induced damage in AT2 cells with PI3K/AKT/mTOR pathway stimulation, underscoring its potential therapeutic value in the treatment of lung injuries.

### Endocan attenuated LPS-induced injury in MLE-12 cells via activation of the PI3K/AKT/mTOR pathway

**Figure 3:**
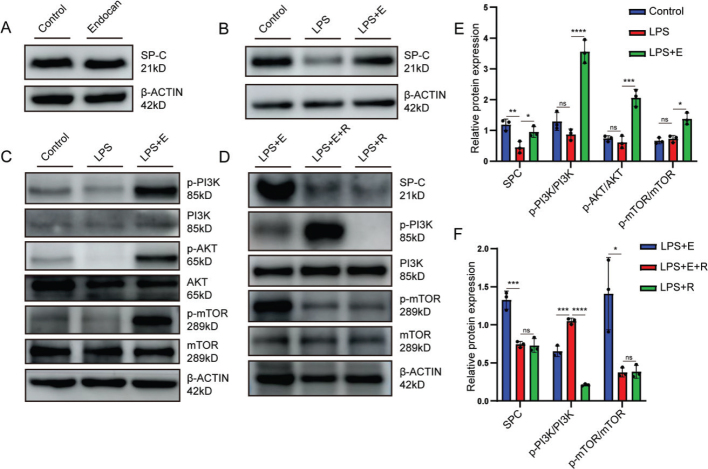
Endocan mitigated LPS-induced epithelial injury and activated the PI3K/AKT/mTOR signaling pathway in MLE-12 cells. MLE-12 cells were divided into six groups: Control, Endocan (30 ng/mL), LPS (10 μg/mL), LPS + Rapamycin (250 nM), LPS + Endocan (30 ng/mL), LPS + Endocan (30 ng/mL) + Rapamycin (250 nM). (A) Western blot analysis showing surfactant protein C (SP-C) expression in Normal and Endocan group. (B) SP-C expression in LPS and LPS + Endocan group. (C) Phosphorylation levels of PI3K, AKT, and mTOR in LPS and LPS + Endocan group. (D) SP-C and PI3K/mTOR phosphorylation levels in cells treated with LPS + Endocan or LPS + Endocan + Rapamycin. (E) Densitometric analysis of grayscale band intensities for the indicated proteins shown in (A, B) (*n* = 3). (F) Densitometric analysis of grayscale band intensities for the indicated proteins shown in (C, D) (*n* = 3). Data are expressed as mean ± standard deviation. One-way analysis of variance (ANOVA) was used to statistical analyze; **P < 0.01, ***P < 0.001, ****P < 0.0001, *P < 0.05.

Given the observed preservation of AT2 cells in lung tissues, we next employed MLE-12 cells as an in vitro model of alveolar epithelial cells. Western blot analysis showed that endocan treatment alone did not significantly alter SP-C expression under basal conditions (Figure 3A). However, upon co-treatment with LPS, endocan partially restored SP-C levels (Figure 3B), suggesting that there existed a protective role against LPS-induced injury. To confirm whether the PI3K/AKT/mTOR signaling pathway mediates the protective effects of endocan, we examined the activation status of key components in this pathway, as well as the impact of pharmacological inhibition using rapamycin. We found that endocan attenuated the LPS-induced reduction in the phosphorylation of PI3K, AKT, and mTOR (Figure 3C). Furthermore, the protective effects of Endocan were abolished by rapamycin, an mTOR inhibitor, as evidenced by decreased SP-C expression and suppression of PI3K/mTOR phosphorylation (Figure 3D). As a consequence, these findings provide cellular-level evidence that endocan protects alveolar epithelial cells against LPS-induced injury through activation of the PI3K/AKT/mTOR pathway, consistent with the in vivo observations.

### Rapamycin diminished the beneficial effects of endocan on AT2 cells in vivo

**Figure 4:**
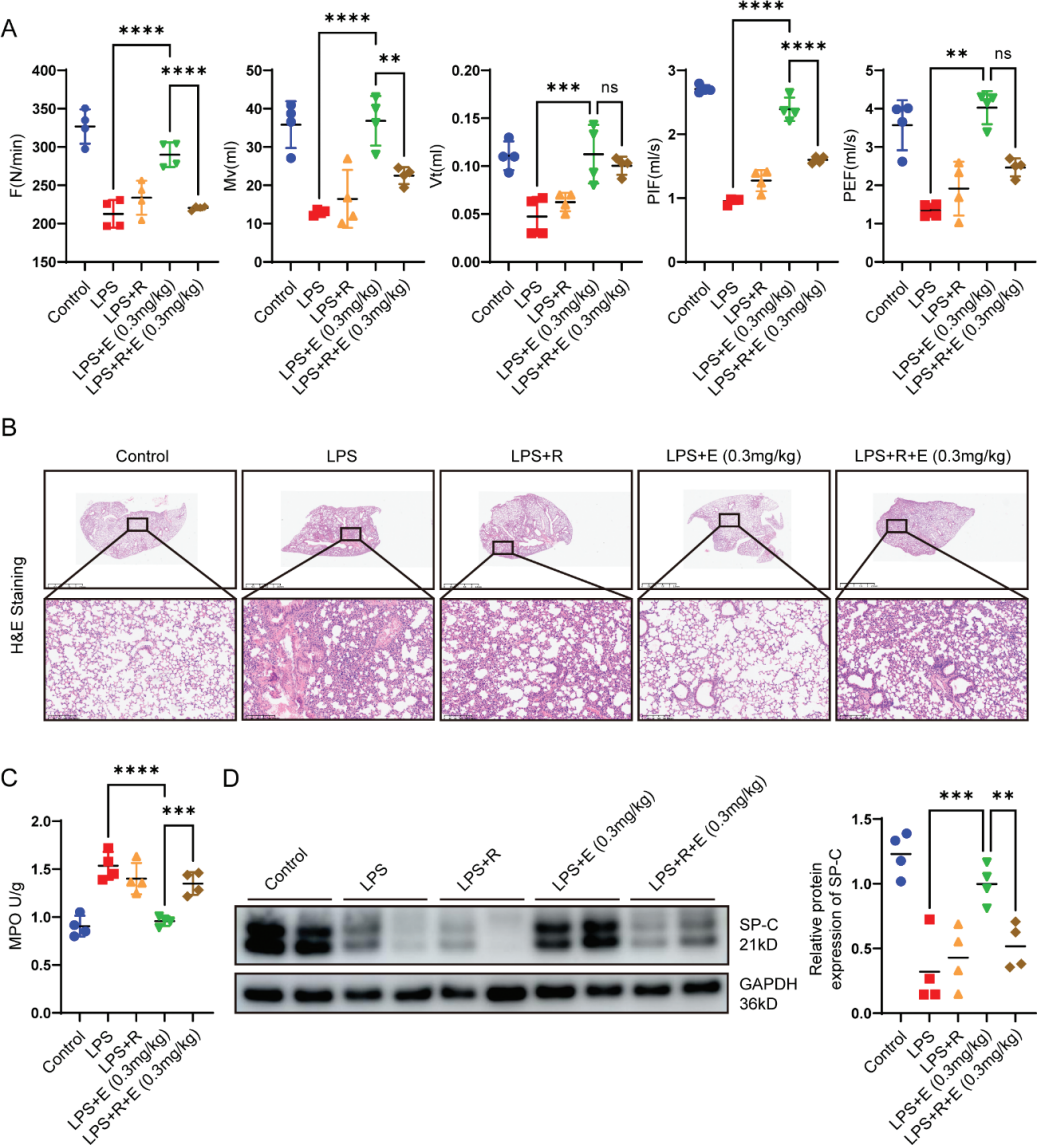
Inhibition of mTOR by rapamycin abrogated the protective effect of endocan on pulmonary function and histopathology. Male C57BL/6 mice were randomized into 5 group: Control, LPS, LPS + Rapamycin (2mg/kg), LPS + Endocan (0.3 mg/kg), and LPS + Endocan (0.3 mg/kg) + Rapamycin (2mg/kg) (*n* = 4). (A) Rapamycin was administered 3 days prior, and pulmonary function, including F, MV, VT, PIF, PEF were assessed 5 h after LPS instillation as described in the methods. (B) Representative hematoxylin and eosin (H&E) staining images of the lung tissues. (C) MPO levels were assessed by corresponding assay kit. (D) Western blot analysis was conducted to assess the expression levels of surfactant protein C (SP-C) in each group. Grayscale statistical analysis was performed to quantify the expression. *n* = 4, data are expressed as mean ± SD. One-way analysis of variance (ANOVA) was used to statistical analyze; **P < 0.01, ***P < 0.001, ****P < 0.0001.

To further validate the role of the PI3K/AKT/mTOR pathway in the protective effects of endocan on AT2 cells in vivo, rapamycin was used to inhibit mTOR activation. Compared to the Endocan group, mice pretreated with rapamycin exhibited a decline in respiratory parameters, including F, Mv, Vt, PEF and PIF (Figure 4A), along with increased MPO levels and exacerbated histopathological changes, such as alveolar wall thickening and heightened inflammatory cell infiltration (Figure 4B and C). Mechanistically, rapamycin pretreatment suppressed the endocan-mediated upregulation of SP-C expression (Figure 4D) and significantly inhibited the activation of the PI3 K/AKT/mTOR signaling pathway (Figure S1). To exclude the potential effects of rapamycin, we contrasted the Rapamycin group with the Control group and found no significant differences in lung function, histopathological features and the expression levels of inflammatory cytokines TNF-α and IL-6 (Figure S2). These findings confirm that rapamycin alone did not induce any significant alterations in lung tissue structure or function. These findings confirm that the PI3K/AKT/mTOR pathway is a critical mechanism through which endocan protected AT2 cells from LPS-induced ALI.

## Discussion

ALI is a serious respiratory disease that currently lacks effective treatment options (25). Endocan, a 50 kDa proteoglycan primarily expressed in the renal and pulmonary endothelium, is considered a novel biomarker of endothelial cell function (26). Researches on respiratory disorders, especially ALI and ARDS, have shown a strong association between circulating endocan levels and disease severity (12, 27, 28). Notably, patients with ALI or respiratory failure exhibit significantly reduced serum endocan levels, suggesting its potential as a prognostic biomarker for these conditions (9). In addition, studies have shown that endocan can inhibit the interaction between lymphocyte function-associated antigen 1 (LFA-1) and intercellular adhesion molecule 1 (ICAM-1), thereby modulating leukocyte adhesion and tissue infiltration (29, 30). Our previous study demonstrated that endocan could significantly suppress inflammatory responses in ALI by attenuating UPRmt-associated apoptosis and modulating cellular bioenergetics. Building on these evidences, our study aimed to further explore whether endocan protects AT2 cells and mitigates lung injury through activation of the PI3K/AKT/mTOR signaling pathway.

In this study, we established LPS-induced ALI mice model and treated it with endocan. Our results demonstrated that endocan markedly alleviated LPS-induced pulmonary function impairment. Given the central role of AT2 cells in surfactant production and alveolar repair, we further investigated the impact of endocan on AT2 cell function. Lung surfactant is a lipid–protein complex composed of approximately 90% lipids, with phosphatidylcholine as the principal component, along with four specific surfactant proteins: SP-A, SP-B, SP-C, and SP-D (31, 32). SP-C, predominantly synthesized by AT2 cells, plays a vital role in reducing surface tension at the air–liquid interface within the alveoli, thereby preventing lung collapse during the ventilatory cycle (33, 34). In our study, endocan treatment led to a marked improvement in LBs, characterized by more defined and organized lamellar layers following LPS administration. These morphological improvements were accompanied by increased SP-C expression, as confirmed by both Western blot and immunofluorescence analysis. Consistently, in the MLE-12 cell line, endocan also restored LPS-suppressed SP-C expression, further supporting that endocan supports AT2 cell function under inflammatory conditions.

To investigate the underlying mechanisms, we focused on the PI3K/AKT/mTOR signaling pathway, a crucial regulator of cell growth and metabolism that govern various physiological functions including cell proliferation, protein synthesis, metabolism, and autophagy (24, 35). MicroRNA-107 was found to alleviate ferroptosis of HK-2 cells by regulating the PI3K/Akt/mTOR pathway in acute kidney injury (36). In osteoporosis and osteolysis, alendronate inhibits autophagy via activation of the TLR4-MyD88/PI3K-AKT-mTOR pathway, in which the impairment prevents the clearance of dysfunctional mitochondria, thereby sustaining superoxide production (37). Wu et al. also demonstrated that exposure to particulate matter enhanced macroautophagy/autophagy in human bronchial epithelial cells and mouse airway epithelium by inactivating mTOR, leading to impaired lysosomal activity and subsequent airway inflammation (38). As a consequence, we hypothesized that endocan mitigates LPS-induced epithelial injury targeting AT2 cells through the PI3K/AKT/mTOR pathway. Subsequent Western blot analysis measuring phosphorylation levels of PI3K, AKT and mTOR as well as rapamycin inhibition assays further confirmed the involvement of the PI3K/AKT/mTOR pathway.

However, given that our findings indicate that endocan protects AT2 cells by activating the PI3K/AKT/mTOR pathway to alleviate lung injury, the role of the PI3K/AKT/mTOR pathway in ALI still remains contradictory across studies. For instance, Xie et al. found that LPS activates the PI3K/Akt/mTOR pathway, promoting the proliferation of mouse lung fibroblasts and the progression from ALI to ARDS (39), while Hu et al. similarly concluded that this activation exacerbates lung injury by promoting collagen synthesis (40). Conversely, Qiao et al. reported that the PI3K/Akt pathway can improve ALI by regulating macrophage M2a polarization (41). These findings suggest that the activation of PI3K/Akt/mTOR may be compartmentalized among different cell types, leading to varied disease outcomes.

Our findings highlight the importance of PI3K/Akt/mTOR pathway in preserving AT2 cell integrity and further support the notion that AT2 cells represent a primary target of endocan in the context of ALI. However, several limitations should be acknowledged. Firstly, only a single early time point (6 h) was analyzed, which mainly reflects the acute inflammatory phase rather than the full spectrum of ARDS progression (42). Secondly, although rapamycin was applied to inhibit mTOR signaling, its action is primarily directed toward mTORC1, and the potential contribution of mTORC2 remains to be clarified (43). In addition, in vitro experiments were conducted using MLE-12 cells instead of primary AT2 cells, which may not fully recapitulate the in vivo characteristics of AT2 cells (44). Future studies incorporating multiple observation time points, and validation in primary cell models are warranted to substantiate and extend these findings.

In conclusion, endocan protects AT2 cells to attenuate LPS-induced lung injury by activating the PI3K/Akt/mTOR pathway. These findings elucidate a novel cell-specific protective mechanism and suggest potential clinical utility of endocan as a therapeutic target in ALI.

## Supplementary Material



## Data Availability

The datasets generated and/or analysed during this study are available in the [jianguoyun] repository, (https://www.jianguoyun.com/p/DWKorTUQ2sz_DBimnOcFIAA).

## Authors’ information

Zekai Wang, Yulu Zhao and Ming Luo are the co-first authors.

## References

[1] Xia L, Zhang C, Lv N, Liang Z, Ma T, Cheng H, et al. AdMSC-derived exosomes alleviate acute lung injury via transferring mitochondrial component to improve homeostasis of alveolar macrophages. Theranostics. 2022;12(6):2928–47. doi: 10.7150/thno.69533 35401830 PMC8965475

[2] Zhang J, Zhang M, Zhu QM, Xu XR, Feng YL, Lin S, et al. Allosteric regulation of Keap1 by 8β-hydroxy-α-cyclocostunolide for the treatment of acute lung injury. Acta Pharm Sin B. 2024;14(9):4174–8. doi: 10.1016/j.apsb.2024.06.025 39309504 PMC11413700

[3] Mokrá D. Acute lung injury – from pathophysiology to treatment. Physiol Res. 2020;69:S353–S66. doi: 10.33549/physiolres.934602 33464919 PMC8603709

[4] Paris AJ, Hayer KE, Oved JH, Avgousti DC, Toulmin SA, Zepp JA, et al. STAT3-BDNF-TrkB signalling promotes alveolar epithelial regeneration after lung injury. Nat Cell Biol. 2020;22(10):1197–210. doi: 10.1038/s41556-020-0569-x 32989251 PMC8167437

[5] Sha HX, Liu YB, Qiu YL, Zhong WJ, Yang NS, Zhang CY, et al. Neutrophil extracellular traps trigger alveolar epithelial cell necroptosis through the cGAS-STING pathway during acute lung injury in mice. Int J Biol Sci. 2024;20(12):4713–30. doi: 10.7150/ijbs.99456 39309425 PMC11414388

[6] Chen X, Zhang C, Wei T, Chen J, Pan T, Li M, et al. α7nAChR activation in AT2 cells promotes alveolar regeneration through WNT7B signaling in acute lung injury. JCI Insight. 2023;8(15):e162547. doi: 10.1172/jci.insight.162547 37410546 PMC10445688

[7] Scherpereel A, Gentina T, Grigoriu B, Sénéchal S, Janin A, Tsicopoulos A, et al. Overexpression of endocan induces tumor formation. Cancer Res. 2003;63(18):6084–9. 14522939

[8] Lassalle P, Molet S, Janin A, Heyden JV, Tavernier J, Fiers W, et al. ESM-1 is a novel human endothelial cell-specific molecule expressed in lung and regulated by cytokines. J Biol Chem. 1996;271(34):20458–64. doi: 10.1074/jbc.271.34.20458 8702785

[9] Hureau M, Portier L, Prin M, De Nadai P, Balsamelli J, Tsicopoulos A, et al. Evaluation of endocan as a treatment for acute inflammatory respiratory failure. Cells. 2023;12(2):257. doi: 10.3390/cells12020257 36672192 PMC9857156

[10] Zhang SM, Zuo L, Zhou Q, Gui SY, Shi R, Wu Q, et al. Expression and distribution of endocan in human tissues. Biotech Histochem. 2012;87(3):172–8. doi: 10.3109/10520295.2011.577754 21526908

[11] Zheng X, Soroush F, Long J, Hall ET, Adishesha PK, Bhattacharya S, et al. Murine glomerular transcriptome links endothelial cell-specific molecule-1 deficiency with susceptibility to diabetic nephropathy. PLoS One. 2017;12(9):e0185250. doi: 10.1371/journal.pone.0185250 28934365 PMC5608371

[12] De Freitas Caires N, Gaudet A, Portier L, Tsicopoulos A, Mathieu D, Lassalle P, et al. Endocan, sepsis, pneumonia, and acute respiratory distress syndrome. Crit Care. 2018;22(1):280. doi: 10.1186/s13054-018-2222-7 30367649 PMC6204032

[13] Zhang X, Zhuang R, Wu H, Chen J, Wang F, Li G, et al. A novel role of endocan in alleviating LPS-induced acute lung injury. Life Sci. 2018;202:89–97. doi: 10.1016/j.lfs.2018.04.005 29627442

[14] Orbegozo D, Rahmania L, Irazabal M, Mendoza M, Annoni F, De Backer D, et al. Endocan as an early biomarker of severity in patients with acute respiratory distress syndrome. Ann Intensive Care. 2017;7(1):93. doi: 10.1186/s13613-017-0311-4 28884313 PMC5589715

[15] Ying J, Zhou D, Gu T, Huang J. Endocan, a risk factor for developing acute respiratory distress syndrome among severe pneumonia patients. Can Respir J. 2019;2019:2476845. doi: 10.1155/2019/2476845 31065299 PMC6466887

[16] Yu L, Wei J, Liu P. Attacking the PI3K/Akt/mTOR signaling pathway for targeted therapeutic treatment in human cancer. Semin Cancer Biol. 2022;85:69–94. doi: 10.1016/j.semcancer.2021.06.019 34175443

[17] Lefranc F, Brotchi J, Kiss R. Possible future issues in the treatment of glioblastomas: special emphasis on cell migration and the resistance of migrating glioblastoma cells to apoptosis. J Clin Oncol. 2005;23(10):2411–22. doi: 10.1200/JCO.2005.03.089 15800333

[18] Wang C, Zou K, Diao Y, Zhou C, Zhou J, Yang Y, et al. Liensinine alleviates LPS-induced acute lung injury by blocking autophagic flux via PI3K/AKT/mTOR signaling pathway. Biomed Pharmacother. 2023;168:115813. doi: 10.1016/j.biopha.2023.115813 37922654

[19] Feng T, Zhou L, Gai S, Zhai Y, Gou N, Wang X, et al. Acacia catechu (L.f.) Willd and Scutellaria baicalensis Georgi extracts suppress LPS-induced pro-inflammatory responses through NF-кB, MAPK, and PI3K-Akt signaling pathways in alveolar epithelial type II cells. Phytother Res. 2019;33(12):3251–60. doi: 10.1002/ptr.6499 31506998

[20] Gaudet A, Portier L, Prin M, Copin MC, Tsicopoulos A, Mathieu D, et al. Endocan regulates acute lung inflammation through control of leukocyte diapedesis. J Appl Physiol (1985). 2019;127(3):668–78. doi: 10.1152/japplphysiol.00337.2019 31295063

[21] Li N, Liu B, Xiong R, Li G, Wang B, Geng Q. HDAC3 deficiency protects against acute lung injury by maintaining epithelial barrier integrity through preserving mitochondrial quality control. Redox Biol. 2023;63:102746. doi: 10.1016/j.redox.2023.102746 37244125 PMC10199751

[22] Ortiz ME, Thurman A, Pezzulo AA, Leidinger MR, Klesney-Tait JA, Karp PH, et al. Heterogeneous expression of the SARS-coronavirus-2 receptor ACE2 in the human respiratory tract. EBioMedicine. 2020;60:102976. doi: 10.1016/j.ebiom.2020.102976 32971472 PMC7505653

[23] Laloglu E, Alay H. Endocan as a potential marker in diagnosis and predicting disease severity in COVID-19 patients: a promising biomarker for patients with false-negative RT-PCR. Ups J Med Sci. 2022;12:8211. 10.48101/ujms.v127.8211 PMC8788653 35140869

[24] Brown EJ, Albers MW, Shin TB, Ichikawa Ichikawa K, Keith CT, Lane WS, K, Keith CT, Lane WS, et al. A mammalian protein targeted by G1-arresting rapamycin-receptor complex. Nature. 1994;369(6483):756–8. doi: 10.48101/ujms.v127.8211 8008069

[25] Ding Z, Zhong R, Xia T, Yang Y, Xing N, Wang W, et al. Advances in research into the mechanisms of Chinese Materia Medica against acute lung injury. Biomed Pharmacother. 2020;122:109706. doi: 10.1016/j.biopha.2019.109706 31918277

[26] Pan KF, Yang YC, Lee WJ, Hua KT, Chien MH. Proteoglycan endocan: a multifaceted therapeutic target in cancer. Biochim Biophys Acta Rev Cancer. 2022;1877(1):188672. doi: 10.1016/j.bbcan.2021.188672 34953930

[27] Ioakeimidou A, Pagalou E, Kontogiorgi M, Antoniadou E, Kaziani K, Psaroulis K, et al. Increase of circulating endocan over sepsis follow-up is associated with progression into organ dysfunction. Eur J Clin Microbiol Infect Dis. 2017;36(10):1749–56. doi: 10.1007/s10096-017-2988-6 28455780 PMC7101577

[28] Mikkelsen ME, Shah CV, Scherpereel A, Lanken PN, Lassalle P, Bellamy SL, et al. Lower serum endocan levels are associated with the development of acute lung injury after major trauma. J Crit Care. 2012;27(5):522.e11–17. doi: 10.1016/j.jcrc.2011.07.077 PMC3790584 21958978

[29] Gaudet A, Portier L, Mathieu D, Hureau M, Tsicopoulos A, Lassalle P, et al. Cleaved endocan acts as a biologic competitor of endocan in the control of ICAM-1-dependent leukocyte diapedesis. J Leukoc Biol. 2020;107(5):833–41. doi: 10.1002/JLB.3AB0320-612RR 32272492

[30] Li Y, Huang J, Foley NM, Xu Y, Li YP, Pan J, et al. B7H3 ameliorates LPS-induced acute lung injury via attenuation of neutrophil migration and infiltration. Sci Rep. 2016;6:31284. doi: 10.1038/srep31284 27515382 PMC4981866

[31] Haczku A. Protective role of the lung collectins surfactant protein A and surfactant protein D in airway inflammation. J Allergy Clin Immunol. 2008;122(5):861–79; quiz 80–1. doi: 10.1016/j.jaci.2008.10.014 19000577 PMC4097097

[32] Whitsett JA. Review: the intersection of surfactant homeostasis and innate host defense of the lung: lessons from newborn infants. Innate Immun. 2010;16(3):138–42. doi: 10.1177/1753425910366879 20351134

[33] Tang X, Snowball JM, Xu Y, Na CL, Weaver TE, Clair G, et al. EMC3 coordinates surfactant protein and lipid homeostasis required for respiration. J Clin Invest. 2017;127(12):4314–25. doi: 10.1172/JCI94152 29083321 PMC5707157

[34] Keller A, Eistetter HR, Voss T, Schäfer KP. The pulmonary surfactant protein C (SP-C) precursor is a type II transmembrane protein. Biochem J. 1991;277:493–9. doi: 10.1042/bj2770493 1859376 PMC1151261

[35] Mossmann D, Park S, Hall MN. mTOR signalling and cellular metabolism are mutual determinants in cancer. Nat Rev Cancer. 2018;18(12):744–57. doi: 10.1038/s41568-018-0074-8 30425336

[36] Zhao P, Wu Z, Chen H. MicroRNA-107 alleviates ferroptosis-mediated acute kidney injury by regulating the PI3K/Akt/mTOR pathway. BMC Nephrol. 2025;27:61. doi: 10.1186/s12882-024-03913-3 41408178 PMC12821974

[37] Li Y, Liu Y, Shi Z, He M. SIRT3 inhibits autophagy-dependent ferroptosis of HUVECs in the progression of pregnancy-induced hypertension by activating the PI3K/Akt/mTOR axis. Front Mol Neurosci. 2025;18:1620184. doi: 10.3389/fnmol.2025.1620184 41112683 PMC12531148

[38] Wu YF, Li ZY, Dong LL, Li WJ, Wu YP, Wang J, et al. Inactivation of MTOR promotes autophagy-mediated epithelial injury in particulate matter-induced airway inflammation. Autophagy. 2020;16(3):435–50. doi: 10.1080/15548627.2019.1628536 31203721 PMC6999647

[39] Xie T, Xu Q, Wan H, Xing S, Shang C, Gao Y, et al. Lipopolysaccharide promotes lung fibroblast proliferation through autophagy inhibition via activation of the PI3K-Akt-mTOR pathway. Lab Invest. 2019;99(5): 625–33. doi: 10.1038/s41374-018-0160-2 30760865

[40] Hu X, Xu Q, Wan H, Hu Y, Xing S, Yang H, et al. PI3K-Akt-mTOR/PFKFB3 pathway mediated lung fibroblast aerobic glycolysis and collagen synthesis in lipopolysaccharide-induced pulmonary fibrosis. Lab Invest. 2020;100(6):801–11. doi: 10.1038/s41374-020-0404-9 32051533

[41] Qiao X, Wang H, He Y, Song D, Altawil A, Wang Q, et al. Grape seed proanthocyanidin ameliorates LPS-induced acute lung injury by modulating M2a macrophage polarization via the TREM2/PI3K/Akt pathway. Inflammation. 2023;46(6):2147–64. doi: 10.1007/s10753-023-01868-5 37566293 PMC10673742

[42] Domscheit H, Hegeman MA, Carvalho N, Spieth PM. Molecular dynamics of lipopolysaccharide-induced lung injury in rodents. Front Physiol. 2020;11:36. doi: 10.3389/fphys.2020.00036 32116752 PMC7012903

[43] Lee CH, Inoki K, Guan KL. mTOR pathway as a target in tissue hypertrophy. Annu Rev Pharmacol Toxicol. 2007;47:443–67. doi: 10.1146/annurev.pharmtox.47.120505.105359 16968213

[44] Li D, Zhang X, Song Z, Zhao S, Huang Y, Qian W, et al. Advances in common in vitro cellular models of pulmonary fibrosis. Immunol Cell Biol. 2024;102(7):557–69. doi: 10.1111/imcb.12756 38714318

