# Supplementary material for: Endocan attenuates LPS-induced alveolar type II cells injury through PI3K/Akt/mTOR pathway
Source: Ups J Med Sci. 2026 Mar 4;131:10.48101/ujms.v131.13337. doi: 10.48101/ujms.v131.13337 (PMC13054927; doi:10.48101/ujms.v131.13337)
Supplement: Supplementary file 1 [file UJMS-131-13337-s1.pdf]

Supplementary material - Endocan activates PI3K/Akt/mTOR pathway to protect type II alveolar cells against LPS-induced acute lung injury

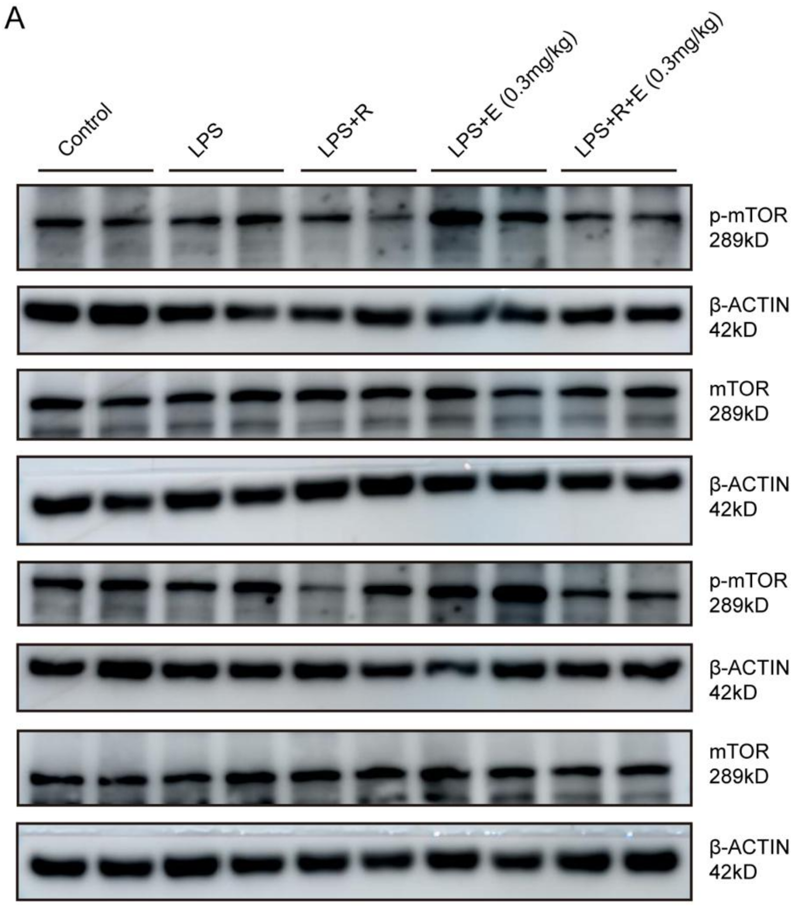

Figure S1: Rapamycin pretreatment inhibits endocan-induced activation of the PI3K/AKT/mTOR signaling pathway.

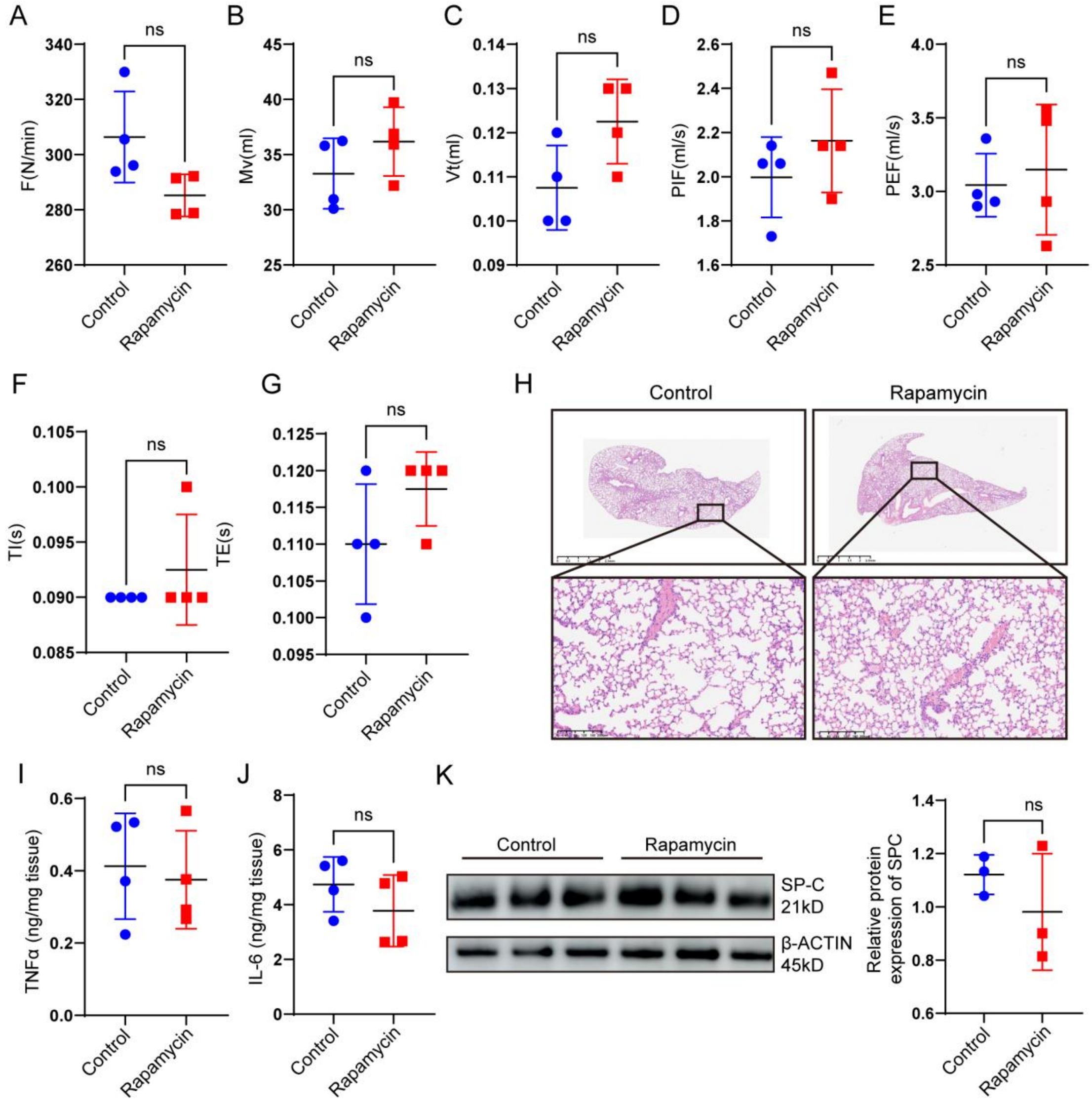

Figure S2: Rapamycin alone does not affect lung function, inflammation, or alveolar epithelial integrity. (A–G) Measurements of lung function parameters in Control and Rapamycin groups. (H) Representative H&E staining of lung tissue sections. (I, J) ELISA analysis of pro-inflammatory cytokines TNF-α and IL-6. (K) Western blot analysis of SP-C expression and quantification of SP-C protein levels by densitometry. One-way analysis of variance (ANOVA) was used to statistical analyze. ns: not significant.
